# A study protocol of a randomized controlled study of internet-based cognitive behavioral therapy for adult attention deficit hyperactivity disorder

**DOI:** 10.1016/j.invent.2023.100652

**Published:** 2023-07-21

**Authors:** David Forsström, Martin Oscarsson, Monica Buhrman, Alexander Rozental

**Affiliations:** a Department of Psychology, Uppsala University, Sweden; b Department of Clinical Neuroscience, Karolinska Institutet, Sweden; c Department of Psychology, Stockholm University, Sweden

**Keywords:** Attention deficit hyperactivity disorder, Internet-based cognitive behavioral therapy, Randomized controlled trial, Quantitative, qualitative

## Abstract

Attention deficit hyperactivity disorder (ADHD) is a disorder characterized by hyperactivity, impulsivity and lack of attention. It can have a major impact on everyday life and result in negative consequences for one's personal, academic, and work situation. For individuals with symptoms of ADHD, increased levels of anxiety and depression are common, and an overall reduction of quality of life is often present. This study protocol describes a clinical trial of internet-based cognitive behavioral therapy (iCBT), using a randomized controlled study design, with the primary aim to increase quality of life, as well as to reduce symptoms of ADHD, anxiety, depression, and stress. A second aim is to investigate, by qualitative means, what aspects of treatment were perceived as helpful and hindering when it comes to completing iCBT. Two hundred participants with symptoms of ADHD will be included and randomized to two conditions (treatment and wait-list control). The treatment period is comprised of a maximum of ten weeks, with two mandatory modules and ten modules from which the participants can choose freely. Self-report measures will be completed by the participants at baseline and end of treatment, as well as at a six-month follow-up. The treatment is guided by therapists and consists of weekly correspondence with the participants. The study will utilize an intention to treat design, with ANOVAs and Reliable Change Index to evaluate treatment effects. The qualitative part of the project will be interview-based and employ thematic analysis. Lastly, a psychometric evaluation of a common instrument for determining ADHD-symptoms will also be made. The results will hopefully contribute to the evidence base for iCBT for individuals with symptoms of ADHD and help disseminate potentially effective interventions.

## Highlights

• This study investigates the efficacy of ICBT for individuals with symptoms of ADHD.
• Symptoms of ADHD, quality of life, and other psychiatric symptoms will be assessed.
• A six-month follow-up allows an assessment of long-term outcomes.
• Qualitative interview will explore experiences of the treatment and its effects.

## 1 Introduction

Attention deficit hyperactivity disorder (ADHD) is a condition that is characterized by symptoms of hyperactivity, impulsivity and lack of attention. The disorder can cause serious impairments for individuals in their everyday life and functioning (Kooij et al., 2010). The disorder is believed to be caused by genetic and neurological factors, with variance in symptom expression as a result of environmental factors (Barkley, 2015). Worldwide estimated adult prevalence is between two and 5 % (e.g., (Kessler and Adler, 2006; Simon et al., 2009). While national and international guidelines consistently advocate for the treatment of adult ADHD to be multidisciplinary (e.g., Kooij et al. (2019), in practice, pharmacotherapy may be the treatment of choice to aid attention and concentration (Dodson, 2005). Regardless of medication, individuals with ADHD-symptoms can still experience persistent impairment in certain contexts. One such environment is educational settings, particularly studying at a university (Gudjonsson et al., 2009). Most of the educational programs and courses offered in higher education require individuals to plan and execute curricular activities on their own and with little assistance from others, which is more difficult to manage if one is struggling with symptoms of ADHD. Studying at a university is also characterized by a large degree of freedom, distal deadlines, task aversiveness, and many distracting elements, which can cause procrastination and difficulties engaging in goal-directed behavior for most people in general (Svartdal et al., 2020), and individuals with ADHD-symptoms in particular (Niermann and Scheres, 2014). One way of overcoming the problems associated with ADHD-symptoms in educational settings is to provide some form of learning support, for instance tutoring and coaching. DuPaul et al. (2017) have demonstrated that such help can increase grade point averages. However, this type of assistance is specifically aimed towards curricular activities, while leaving such psychological complaints as anxiety and depression unaddressed.

Another environment in which individuals with ADHD-symptoms may experience difficulties is the working environment. Functioning at work can be impaired by ADHD symptoms in several ways, creating problems carrying out specific tasks, which in turn increases stress levels and affect interpersonal relationships (Polanczyk et al., 2007). Individuals with ADHD-symptoms often have difficulties with organization, prioritizing, and following through on instructions, which negatively impact performance at work (De Graaf et al., 2008). Furthermore, symptoms of ADHD have been found to be related to greater fatigue, burnout, and long-term sick-leave (Combs et al., 2015). Hence, providing support for individuals with symptoms of ADHD might be an effective way not only to prevent some of the difficulties often experienced in occupational settings, but also risk factors that might contribute to or exacerbate mental distress (Ek and Isaksson, 2013; Oscarsson et al., 2022).

In addition to pharmacotherapy, psychological treatments may prove beneficial in helping individuals with ADHD-symptoms manage problems in different contexts as well as co-existing psychological complaints (Lambez et al., 2020). However, few studies have examined the effects of psychological treatments for individuals ADHD-symptoms. A recent systematic review suggest that Cognitive Behavior Therapy (CBT) has strong empirical support, with positive effects on measures of both primary (symptoms) and secondary (psychosocial) adult ADHD outcomes, but that the number of studies are few and suffer from methodological issues (Fullen et al., 2020). Similarly, a meta-analysis also found positive effects of more behaviorally-oriented interventions often included in CBT, e.g., behavioral modification, but concluded that the number of publications is small and further research is warranted (Hodgson et al., 2014).

Ramsay (2010) has proposed a CBT-model adapted for adult ADHD, largely based on Barkley's (2015) model of ADHD as an executive function deficit disorder. Ramsay's model includes consideration of the executive dysfunction, as well as the developmental experiences of ADHD adults. This includes recognizing cognitions related to a history of perceived failures, and self-mistrust. With general consensus regarding required coping strategies and techniques for adults with ADHD (e.g., reducing distractions, using planners, behavioral activation), the main challenge for patients and their therapists is the implementation of these skills in everyday life (Ramsay, 2016).

Meanwhile, psychological treatments for ADHD-symptoms might not always be readily available. One way of overcoming this problem of outreach is to provide online interventions, which has proven to be an effective mean of disseminating aid for many psychiatric disorders (Andersson et al., 2019), but is less explored for ADHD-symptoms (Păsărelu et al., 2020). To date, only one meta-analysis has been carried out, which included six studies and found that internet-based interventions increased attention and social functioning. Still, despite some indications of benefit, the number of studies were few and included small sample sizes (*n*s < 30), highlighting the need for additional research (Shou et al., 2022). Apart from outcome studies, investigations on the feasibility and usability of online interventions and smartphone applications have also been made. Overall, this type of research indicate that such means of delivery are accepted and perceived as user-friendly (Knouse et al., 2022), but that issues of content and engagement are important to consider (Kenter et al., 2022; Nordby et al., 2021). A recent study that explored the feasibility of a proposed online intervention for ADHD-symptoms also demonstrated a high acceptability towards this type of aid, and that 59 % of the participants actually preferred online to face-to-face interventions (Shelton et al., 2022). With additional studies investigating online interventions for ADHD-symptoms under way (Kenter et al., 2021), the interest in developing and disseminating this type of support seem to be growing and could, if found to be effective, help more individuals with ADHD-symptoms deal with everyday life and functioning.

### 1.1 Aims of the study

Given the scarcity of research on psychological treatments for ADHD-symptoms in general, and internet-based interventions in particular, there is a need for additional studies in order to test their efficacy. This is especially true for adequately powered randomized controlled trials with longer follow-up assessments (Shou et al., 2022). Furthermore, given the many challenges that face individuals with ADHD-symptoms in both educational and occupational settings and the lack of support being provided in these contexts, providing online interventions may prove to be helpful for many university students and employed individuals with these types of issues. Thus, the aim of the current study is to conduct a clinical trial examining the effects of an internet-based intervention for individuals with ADHD-symptoms to determine its impact on inattention, restlessness, psychological complaints, and quality of life among university students and individuals working at-least part time in Sweden. Results from a previous Swedish study that guided use of smartphone applications for individuals with ADHD-symptoms implies that online interventions can be accepted and generate benefits in terms of reduction of inattention (Moëll et al., 2015), but further research is needed. In addition, there is also a need to understand the experiences of undergoing such a treatment to find out what aspects of online interventions are most useful, and which might warrant further refinement. The following research questions will be addressed:

### 1.2 Research questions

• What are the group-average outcomes of the online intervention at the end of treatment and at a six-month follow-up?
• How many individuals experience a reliable change when it comes to ADHD-symptoms and quality of life at the end of treatment and at a six-month follow-up?
• Did the participants experience any negative consequences in relation to the online intervention?
• How do participants who have discontinued treatment view the online intervention and their participation?
• How do people who have undergone the entire treatment (≥ six modules) perceive the online intervention and their participation, both in terms of helping and hindering aspects?
• What are the psychometric properties of the Swedish translation of ASRS in a Swedish population?

## 2 Material and methods

### 2.1 Procedure

Participants will be recruited via two contexts; 1) educational settings, using student healthcare centres at different universities in Sweden, and information in emails, advertisements on campus, and social media aimed at university students, and 2) occupational settings, using advertisements on social media. Furthermore, relevant patient organisations will also be contacted and asked to inform their members about the study. In addition, a website where studies of online interventions are advertised will also help out with outreach (www.studie.nu). Those interested in participating will then sign up via a survey hosted at REDCap, i.e., a secure online platform for data collection (Harris et al., 2019), and submit baseline data, i.e., demographics and self-report measures. Informed consent by the participants will be submitted at this stage. Those individuals fulfilling the initial inclusion criteria will then be contacted by members from the research team in order to conduct a structured clinical interview to determine eligibility for inclusion and whether any exclusion criteria are fulfilled in terms of the presence of a severe mental disorder, i.e., the The Mini-International Neuropsychiatric Interview (MINI) (Sheehan et al., 1998). The participants will be allocated into two groups: treatment and control group. The participants in the treatment group will receive information about how to sign up to the treatment platform that is supplied by Psykologpartners W & W AB, a private healthcare provider in Sweden who have developed and manages the online intervention that is being tested in the current study. The participants will undergo a 10-week treatment program targeting common symptoms for individuals with ADHD. The treatment is guided by weekly check-ins by a therapist who receives weekly supervision, i.e., a psychologist in training undergoing basic clinical education in CBT at Uppsala University.

The participants will complete the same survey at the end of the online intervention, with the addition of the Negative Effects Questionnaire (NEQ; Rozental et al., 2019). Participants will also complete a similar survey at a six-month follow up, but this time without the NEQ (see Table 1 for an overview). After the six-month follow-up, the control group will be offered to receive the same online intervention as the treatment group, who in turn will complete the same surveys as described above. Participants in the control condition will respond to a self-report measure (PHQ-9) on depression weekly throughout their participation to monitor any eventual deterioration or suicidal ideation. If a participant receiving the online intervention decides to end treatment prematurely, the participant will be contacted to partake in an interview and asked to still complete the survey given at the end of the treatment period. Meanwhile, participants completing the full online intervention will be asked to partake in an interview about what they experienced as beneficial and hindering about the treatment. For the psychometric evaluation of the ASRS, data from the baseline screening will suffice to answer the research question. The recruitment will commence in February of 2023 and will end in September to October of 2023.

**Table 1:** Overview of the self-report measures.

Self-report measure	Assessment		
	Baseline	Post-treatment	Six-month follow-up
AAQoL	X	X	X
ASRS	X	X	X
GAD-7	X	X	X
PHQ-9	X	X	X
PSS	X	X	X
NEQ		X	

AAQoL = Adult Attention-deficit/hyperactivity disorder Quality-of-Life scale; ASRS = Adult ADHD Self-Report Scale-V1.1 (ASRS-V1.1) Symptoms Checklist; GAD-7 = Generalized Anxiety Disorder; PHQ-9 = Patient Health Questionnaire; PSS = Perceived Stress Scale; NEQ = Negative Effects Questionnaire.

### 2.2 Participants

**Fig. 1:**
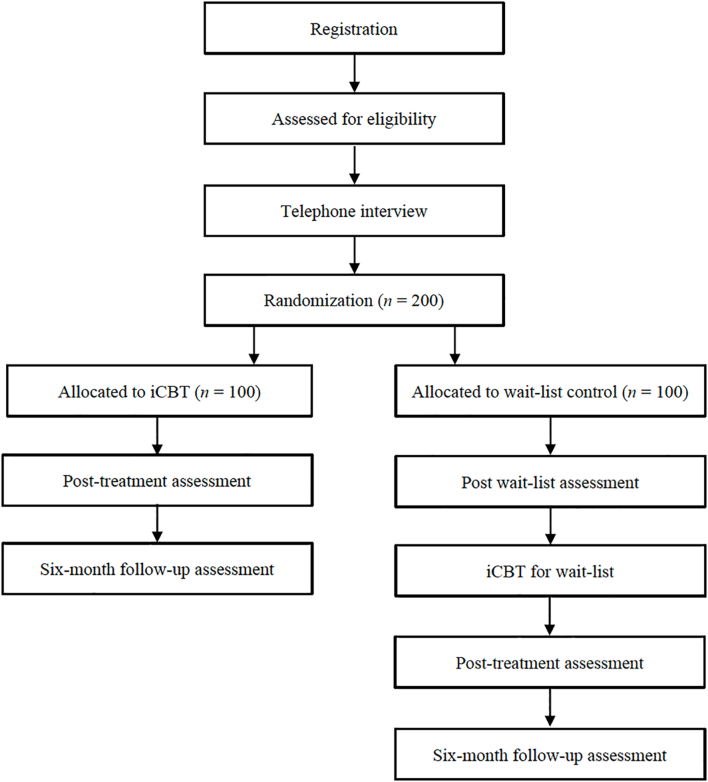
Flow chart for the current study.

A power analysis was conducted using G*Power 3.1 to ascertain the number of participants needed to detect differences at post treatment and follow-up. The sample size calculation was based on three time points: pretreatment, post treatment, and at a six-month follow-up for both groups. The anticipated effect size of the treatment was set to Cohen's d 0.1 and the analysis used was a repeated measures ANOVA with Tukey's post hoc test. The randomization is carried out via www.random.org using space noise a randomization principle. The allocation will be into two groups: treatment and waitlist control group. No concealment procedure was used since the participants in the control group were informed that they would receive treatment at a later stage. The low effect was set based on the fact that this type of treatment has not been thoroughly researched and several existing studies have demonstrated a small effect. As a result, the number of participants needed was 138, but it was decided to include 200 participants to account for an expected high dropout rate. As for the qualitative studies, twelve to fifteen participants for each study will be interviewed, which is regarded as adequate to achieve code saturation in the qualitative analysis (Hennink et al., 2017). In terms of the psychometric evaluation of the ASRS (Study 5), two hundred participants are considered sufficient for determining internal consistency, convergent and divergent validity with other self-report measures, and to conduct exploratory and confirmatory factor analyses. See Fig. 1 for a flow chart over the recruitment process.

### 2.3 Inclusion and exclusion criteria

In the current study, the following inclusion criteria will be used when assessing whether an individual can get access to psychological treatment:

1) Over 18 years old of age.
2) Can read and write in Swedish.
3) Have access to a computer, tablet, or smartphone with an Internet connection.
4) Have previously been diagnosed with ADHD.
5) Are studying at a university or college in Sweden or working at least 50 % of a full time-employment.

The following exclusion criteria will be used:

1) Shows elevated symptoms of depression or risk of suicide, i.e., a total score on the Patient Health Questionnaire – 9 items ((PHQ-9; Kroenke et al. (2001), >15 points, or scores >2 points on question i) concerning suicidality.
2) Has started or changed their medication for any psychiatric diagnosis in the last three months.
3) Exhibits another form of psychiatric diagnosis that requires more specialized care, for example substance abuse syndrome, anorexia nervosa, bipolar disorder, psychotic symptoms, or schizophrenia, as assessed with the MINI (Sheehan et al., 1998). There are no additional exclusion criteria for the qualitative studies regarding dropouts or participants that completed the treatment.

### 2.4 Withdrawal

Participants can withdraw from the current study at any moment. The reason for this will be explored for research purposes, but the individual will be informed that he/she can defer from specifying her motive.

### 2.5 Self-report measures

#### 2.5.1 Adult Attention-deficit/hyperactivity disorder Quality-of-Life scale (AAQoL)

The AAQoL (Brod et al., 2015; Brod et al., 2006) is a disease-specific quality of life measure for adults with ADHD. The instrument measures quality of life-consequences of ADHD in four domains using and consists of 29 items. The instrument has excellent internal consistency (Cronbach's α = 0.93 for the overall measure). One example of an item is “In the last two weeks, how hard has it been for you to remember important things?”, scored on a five-point Likert-like scale from “Not at all/Never” (1) to “Extremely/Very Often” (5). The scale ranges from 0 to 100. The sum of the AAQoL is one of the two main outcomes measures in the study.

#### 2.5.2 Adult ADHD Self-Report Scale-V1.1 (ASRS-V1.1) Symptoms Checklist (ASRS)

The ASRS is an instrument that measures ADHD-symptoms (Kessler et al., 2005). The instrument consists of 18 items that correspond to the 18 symptom-criteria for ADHD found in the DSM-IV-TR (Association, 2000). Of these 18 questions, six have been identified as most predictive of ADHD-symptoms. The overall range of the entire measure is 0 to 72. However, there are separate items focusing on attention and hyperactivity. The ASRS is the second main outcome measure of the study.

#### 2.5.3 Generalized Anxiety Disorder – 7 Items (GAD-7)

The GAD-7 is an instrument that measures anxiety (Spitzer et al., 2006). Its internal consistency is excellent (Cronbach's α = 0.92), and it has good test-retest reliability (IntraClass Correlation = 0.83; Spitzer et al., 2006). The instrument includes seven items and has a single-factor solution. The score ranges from 0 to 21 points and the items range from 0 (“Not at all”) to 3 (“Nearly every day”; Spitzer et al., 2006). One example of an item is: “Over the last 2 weeks, how often have you been bothered by any of the following problems? Feeling nervous, anxious, or on edge.” (Item 1).

#### 2.5.4 Patient Health Questionnaire – 9 Items (PHQ-9)

The PHQ-9 is a nine-item instrument measuring depression. It has excellent internal consistency (Cronbach's α = 0.89) and a good test-retest correlation (*r* = 0.84; Kroenke et al., 2001). The instrument has a single-factor solution. The score ranges from 0 to 27 points (Kroenke et al., 2001). Items range from 0 (“Not at all”) to 3 (“Nearly every day”). One example of an item is: “Over the last 2 weeks, how often have you been bothered by any of the following problems? Little interest or pleasure in doing things.” (Item 1).

#### 2.5.5 Perceived Stress Scale (PSS)

The PSS is an instrument that evaluates the subjective experience of general stress in various situations and is scored on a five-point Likert-scale 0–4 (“Never” to “Very often”), with seven items being scored in reverse (items 4–7, 9–10, and 13). The PSS includes 14 items in total and has been shown to have good internal consistency (Cronbach's α = 0.84–0.86) as well as good convergent and discriminant validity (Cohen et al., 1983).

#### 2.5.6 Negative Effects Questionnaire (NEQ)

The NEQ is a 32-item instrument that assesses unwanted and adverse events experienced by patients undergoing psychotherapy (Rozental et al., 2016; Rozental et al., 2019), e.g., “I experienced more unpleasant feelings” (Item 11). It is comprised of six factors: symptoms, quality, dependency, stigma, hopelessness, and failure. The internal consistency for the full instrument is excellent (Cronbach's α = 0.95), and is scored on several dimensions; 1) “Did you experience this?” (yes/no), 2) “If yes – here is how negatively it affected me”, from 0 (“Not at all”) to 4 (“Extremely”), and 3) “Probably caused by” (“The treatment I received” or “Other circumstances”).

### 2.6 Treatments and therapists

The online treatment will be administered over ten weeks and consists of two mandatory modules and ten modules that can be selected from. The participants need to carry out the two mandatory modules and at least four additional modules that they have chosen to complete the treatment. The modules target different aspects of everyday functioning (e.g., how to plan your day and how to avoid losing things).

The intervention is inspired by the mechanisms of change in CBT for adult ADHD proposed by Ramsay (2010), including rudimentary mental scaffolding through psychoeducation, and ADHD-friendly environmental engineering to mitigate the consequences of executive dysfunction. The intervention is partly individualized through the therapist support, which also focuses on implementation strategies for participants to turn intentions into action (Ramsay, 2015). The therapists will have weekly check-ins with the patients in order to provide guided support. If a patient does not log in for a week the assigned therapist will send a SMS as reminder to log in and if the patient does not log in for two weeks, the assigned therapist will call the patient.

There are five basic modules to choose from and every basic module also has an advanced module (containing more tools and information) that can be selected after the completion of the basic module. The participants can choose a maximum of four basic modules and four advanced modules during treatment. See Table 2 for more information.

**Table 2:** Treatment content.

Basic treatment modules
Introduction module This module provides psychoeducation about ADHD and introduces the patient to the program.
“I have a hard time scheduling my time” This module focuses on planning and dealing with time in different ways to make it easier for everyday planning.
“I have a hard time maintaining everyday routines” This module focuses on establishing and maintaining routines to simplify everyday life.
“I often loose things” This module focuses on tools to help keep track and remember things, for example by categorization and organization. Work with acceptance is also a part of this module.
“I often feel stressed” This module focuses on strategies to better handle stress by both going back to our basic needs (food, sleep, and exercise) as well as looking at other skills and tools regarding stress.
“I have a hard time staying focused” This module focuses on attention and provides different tools to make it easier to maintain attention in different situations.
Exit module In the exit module, the treatment is summarized, and the patient creates a plan forward. The module also touches upon what to do if things get hard or the tools are forgotten.

The therapists will be university students undergoing basic clinical training in CBT during their final year at the Psychology Program at Uppsala University. They will have completed two to three semesters of clinical training when the current study is planned to commence. The therapists will receive a two-day training in the specific online CBT that will be administered in the study, provided by Psykologpartners W & W AB. The therapists will also receive weekly supervision by experienced clinical psychologists who developed the online intervention.

### 2.7 Ethics and registration

The ethics application (Dnr. 2022-06261-01) was approved by the Swedish Ethical Review Authority and has been registered at ClinicalTrials.gov (NCT05700539). Participants will provide informed consent during the recruitment process. Great consideration will also be made to prevent participants faring worse by monitor their condition, i.e., deterioration in the control group is monitored by the researchers and deterioration in the treatment group is monitored by the therapists. Meanwhile unwanted and adverse events will be explored after treatment has ended.

### 2.8 Statistical analysis

All outcomes will be analyzed using a series of ANOVAs, according to intent-to-treat-principle and missing values will be handled by using multiple imputation. Bonferroni-corrections to adjust for family-wise errors will also be used. The only exception applies to the NEQ which is only reported descriptively. Individual changes on the AAQoL and ASRS are determined using the Reliable Change Index (Jacobson and Truax, 1992; Maassen, 2004), i.e., yes/no, for improvement, deterioration, and non-response. For recovery, the Reliable Change Index and a positive change of two standard deviations in the direction of functionality will be used, i.e., criterion a (Jacobson and Truax, 1992). Given the large sample size that the study aims to recruit, moderation analyses are possible to conduct, but will be performed post hoc. Exploratory and confirmatory factor analyses will be used alongside reliability calculations (Omega) and analytic techniques used in Rasch analysis.

### 2.9 Qualitative interviews and analysis

For the qualitative studies, participants are recruited based on either dropping out of treatment prematurely or completing the full online intervention. Recruitment is therefore based on participants sharing a specific set of experiences, i.e., critical cases. Thematic analysis will then be applied to explore recurrent themes in their accounts of undergoing treatment (Braun and Clarke, 2006, Braun and Clarke, 2013; Clarke et al., 2015).

## 3 Discussion

The current protocol has described a clinical trial that will investigate the effect of iCBT for individuals with ADHD-symptoms who are either studying at a university or college, or of an adult working population. There is presently a lack of research that has explored the effects of psychological treatments for these particular groups. In addition, there are also few studies investigating the potential in disseminating online interventions for the type of difficulties often associated with symptoms of ADHD. The results from the current study will therefore provide useful knowledge on whether this support could be helpful for adults with ADHD. By combining measures of quality of life and symptoms of ADHD with measures of depression, anxiety and perceived stress, the current study will evaluate the effects of the program on both primary and secondary adult ADHD outcomes, including a six-month follow-up. Subscale scores on the AAQoL at baseline, post-treatment and follow-up could be explored to assess impairment and change in different life domains. There is also a possibility to explore the relationship between AAQoL and ASRS scores throughout the study process. Furthermore, the use of qualitative interviews will enable an investigation on the experiences of undergoing and completing iCBT for ADHD-symptoms, focusing on factors related to both dropout and completion of treatment, which in turn can be useful in developing more effective online interventions. Among the many strengths of the current study is the fact that the treatment will consist of several modules that the participants can choose from, which is believed to match their individual difficulties and settings better than a generic outline. In addition, the size of the sample is much larger compared to previous studies, which will allow more robust statistical inferences and subgroup analyses, as well as a psychometric evaluation of a common instrument for determining ADHD-symptoms. Furthermore, the use of a structured clinical interview to screen participants will provide a better overview of comorbidities among the included participants. Lastly, all of the researchers involved in the current study, as well as Psykologpartners W& W AB who manages the online intervention, have extensive experience of conducting research on iCBT. This contributes to, what we believe, a successful clinical trial in terms of recruitment, treatment delivery, and data collection and analysis. However, there are also a number of limitations that need to be addressed. The use of waitlist controls limits the conclusions that can be inferred from the treatment outcome but was deemed most appropriate given the use of an internet-based modality (i.e., providing a face-to-face treatment as a comparator was not feasible). Moreover, the relatively short follow-up period and the use of few measurement points could limit the inferences that are possible to make about changes during treatment and its long-term effects. However, due to the difficulties facing many individuals with ADHD-symptoms, such as inattention and restlessness, it was not considered possible to include additional measurement points than the ones included. To get the participants to answer the surveys at the pre-determined measure points reminders will be utilized and the therapists will reach out to those not responding in time. Similarly, there is also a risk that many of the participants will drop out from treatment. This will be managed by contacting participants that fail to complete the modules on a weekly basis and offering support in case problems arise. Further, the use of self-referrals in clinical trials always poses a problem since they might not accurately reflect the demographic composition of the target group. This can in turn result in selection bias, which limit the generalizations possible to make from the study. Comparisons to other research samples will therefore be made to understand whether the recruited participants differ in any sense and if this might have affected the results of treatment. Lastly, even though adherence can be monitored with regard to the number of modules completed, it is still difficult to determine the amount of time each participant spent on engaging with the content and the exercises included. The current study will however only be able to probe for this using self-reports and not objective data, which can be seen as a limitation. Furthermore, the fact that ASRS has not been validated in Swedish is a limitation. If the psychometric evaluation does not confirm the instrument's validity this might make it hard to interpret the results of the study. However, ASRS has been used in a several of studies with good results (Kenter et al., 2023; Moëll et al., 2015) and the instrument has been validated with good results in many different languages (Adler et al., 2019; Green et al., 2019; Vňuková et al., 2022; Yeh et al., 2008).

Another limitation is the inclusion criteria regarding university studies and/or employment might create a threshold that excludes individuals with more severe ADHD-symptoms that might have problems with maintaining employment or carrying out university studies. The consequence of this might be that the study fails to recruit a segment of individuals with ADHD that might benefit from treatment.

## 4 Conclusion

The present study protocol has described a clinical trial of iCBT for individuals with symptoms of ADHD and is believed to increase the current understanding of how to provide psychological treatments for a particular group that typically receive very little support apart from pharmacotherapy. The aim is to provide an effective online intervention that might increase access for those who do not receive help, and could, if found beneficial, become an important addition to medication, increasing quality of life and managing symptoms of ADHD, anxiety, depression, and stress.

## Declaration of competing interest

The researchers involved in the current study do not have any economic interests in relation to Psykologpartners W & W AB and have not received any financial support for conducting the research presented.
